# Antioxidants: Mechanisms, Benefits, and the Importance of Extremophilic Microorganisms

**DOI:** 10.3390/microorganisms14040838

**Published:** 2026-04-08

**Authors:** Mohammed Aladhadh

**Affiliations:** Department of Food Science and Human Nutrition, College of Agriculture and Food, Qassim University, Buraydah 51452, Saudi Arabia; aladhadh@qu.edu.sa

**Keywords:** oxidative stress, carotenoids, extremophilic microorganisms, industrial applications, antioxidants

## Abstract

Despite their vital physiological roles, oxidative imbalance caused by reactive oxygen, nitrogen, sulphur, and chlorine species damages essential body macromolecules such as proteins, lipids, and nucleic acids through oxidative stress. This stress is strongly associated with cancer, inflammation, neurological and cardiovascular disorders, and other chronic human diseases. Therefore, antioxidants, natural or synthetic, that counteract oxidative damage are important, with increasing interest in their use within the pharmaceutical, food, and cosmetic industries. However, due to toxicity concerns with the synthetic variants, natural antioxidants are increasingly preferred. Extremophile-derived antioxidants, such as superoxide dismutases, catalases, peroxidases, carotenoids, and melanin, are of renewed interest due to their remarkable stability, robustness, and potency under extreme conditions of temperature, pH, and salinity. These make them better than many mesophile-derived antioxidants and excellent candidates for cost-effective biotechnological, research, and industrial processes that require high operational efficiency. This review summarises key classes of selected enzymatic and pigment antioxidants, their mechanisms of action, and their industrial relevance, with a focus on extremophilic microalgae, bacteria, and fungi. The benefits of extremophilic antioxidants are discussed alongside their current applications and existing challenges, including the need to develop efficient delivery systems, scalability issues, and limited characterisation.

## 1. Introduction

Reactive species such as reactive oxygen species (ROS), reactive sulphur species (RSS), reactive chlorine species (RCS), and reactive nitrogen species (RNS) play key roles in human physiological functions such as cell signalling, immunity, and gene regulation [1,2] and the development of some diseases [3,4]. Reviews of the literature indicate that ROS and other reactive species induce oxidative stress, damaging macromolecules such as proteins, carbohydrates, lipids, and DNA. This damage is thought to contribute to the development of different diseases such as cancer, asthma, cardiovascular problems, arthritis, autoimmune disorders, and neurological problems, including Parkinson’s dementia [2,5]. Substances or compounds that slow down or inhibit the free radical-mediated oxidation of these body macromolecules are called antioxidants [6]. These function by reducing the levels of available reactive species through scavenging, dismutation, and degradation into harmless molecules to mitigate their oxidative damage potential [4].

Antioxidants can be natural or synthetic [4,7]. Natural forms of antioxidants are either exogenous or endogenous. Exogenous antioxidants are those sourced from outside the body, typically through the consumption of plant-based food, herbs, and spices. These include vitamins (e.g., ascorbic acids, vitamins C and E), minerals (e.g., Selenium, copper, manganese, and zinc), carotenoids (e.g., α and β-carotene, lutein, and lycopene), and polyphenols (e.g., flavons, anthocyanins, and phenolic acids) [3,8]. In contrast, endogenous antioxidants are produced naturally in the body via enzymatic and non-enzymatic means. Examples of enzymatic antioxidants include enzymes such as catalase, superoxide dismutase (SOD), and glutathione reductase [8], while low molecular weight compounds such as lipoic acids (lipophilic) and uric acids (hydrophilic), and metal-binding albumin are examples of non-enzymatic antioxidants. Extensive information on the different types of antioxidant systems, sources, mechanisms of action, and potential uses is available [1,4,9].

Synthetic antioxidants are artificial and include recombinant SODs, phenolics, nano-antioxidants (metallic nanoparticles and antioxidant-functionalised nanoparticles), nitrones/nitroxides, iron-ion chelators, and synthetic oxidases [3,4,10]. However, some commonly used synthetic phenolic antioxidants, such as butylated hydroxyanisole (BHA), butylated hydroxytoluene (BHT), and tertiary butylhydroquinone (TBHQ), have been linked to significant adverse health effects, including cytotoxicity, carcinogenicity, and endocrine disruption, making their use controversial [10]. However, due to the observed toxicity, there is increasing interest in the use of antioxidants from natural or biological sources, as these are less toxic, cheaper, and show comparative or greater efficacy than synthetic antioxidants [11,12]. Therefore, this review provides a brief overview of the mechanisms of action of natural and synthetic antioxidants. Thereafter, it focuses on extremophilic microalgae, bacteria, archaea, and fungi as current and emerging sources of robust natural antioxidants, including superoxide dismutases, catalases, peroxidases, carotenoids, and melanin, highlighting their advantages over conventional organisms. The current and potential applications of extremophile-derived antioxidants in the different fields, such as pharmaceutical (medical), food, and industrial sectors, are evaluated alongside existing challenges and research gaps to overcome for broader industrial application.

This review is novel compared to most recent reviews in multiple ways. With respect to scope and coverage, this review treats bacteria, archaea, fungi, and microalgae from different environments as coequal sources of extremophilic antioxidants. This is in contrast to some other reviews or reports that are exclusively focused on antioxidants from a specific group, such as bacteria [13,14], archaea [15], fungi [16], microalgae [17,18,19], or specific genus/species or environment [20]. Therefore, the review presents a cross-kingdom antioxidant–extremophile perspective that allows for ease of comparison and identification of cross-kingdom antioxidants, which is not possible with a single group (e.g., microalgae-only) review. Additionally, while most recent reviews or reports on antioxidants from biological systems are usually limited to one or two sectors (e.g., food or cosmeceuticals) [21,22], this review presents the application of extremophile-derived antioxidants such as SODs, catalases, and carotenoids across *multiple* sectors: food/packaging and preservatives, medicine/pharmaceuticals, cosmetics, textile and manufacturing, and environmental bioremediation. It uniquely identifies gaps in knowledge, delivery challenges, and current industrial applications. Other known antioxidants, such as vitamins E and C, exopolysaccharides, and ectoines, have not been reviewed in this paper.

## 2. Mechanisms of Action of Antioxidants

**Figure 1:**
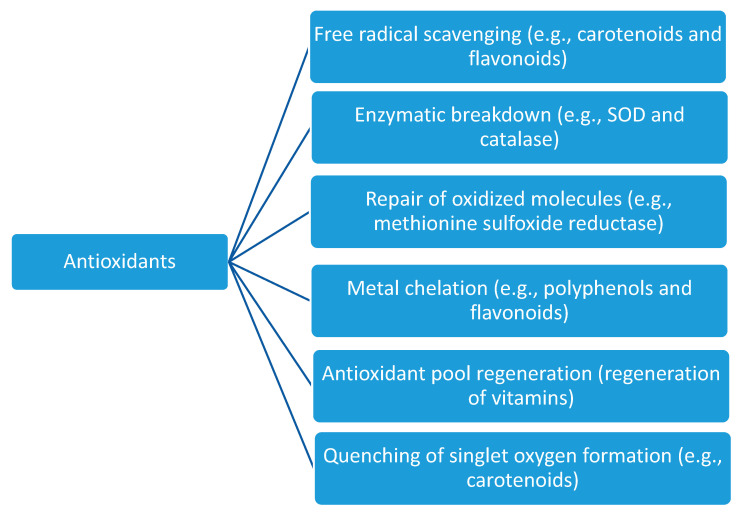
Mode of action of antioxidants.

Antioxidants, whether synthetic or natural, can be categorised into primary antioxidants and secondary antioxidants based on their modes of action. Primary antioxidants directly reduce or inhibit the oxidative processes associated with the reactive species, while secondary antioxidants act indirectly on the oxidative processes [23]. They do this through different means, with primary antioxidants scavenging acting as oxygen scavengers through chain breaking and atom/electron donations, while secondary mechanisms include metal ion chelation, inhibition of peroxides, and singlet oxygen elimination [23,24]. Free radical scavenging is a key mechanism of antioxidant activities. Antioxidants carry this out via two mechanisms: the donation of hydrogen atoms, hydrogen-atom transfer (HAT), or electrons or single-electron transfer (SET) to free radicals, thereby stabilising them before they propagate and initiate oxidative actions [24]. Another mechanism is the use of endogenous enzymatic systems such as superoxide dismutase (SOD), catalase (CAT), and peroxidases to detoxify reactive species. SOD detoxifies oxidants by converting superoxide radicals into hydrogen peroxide and molecular oxygen, while CAT converts hydrogen peroxide into water and oxygen. Other enzymes, such as peroxidases (e.g., glutathione peroxidase, ascorbate peroxidases, and peroxiredoxins), can also detoxify ROS. For example, glutathione peroxidase catalyses the detoxification of hydrogen and lipid peroxides by using reduced glutathione (GSH) to convert these reactive species to water or corresponding alcohols, thereby protecting cells from oxidative damage [25]. Some other antioxidants function through the repair of oxidatively damaged proteins, reversing the effects of the oxidative damage to cells. A good example is methionine sulfoxide reductase. Methionine sulfoxide reductase (Msr) is an example of an antioxidant that repairs damaged or oxidised macromolecules [26]. Msr acts by reversing the reactive species-induced oxidation of methionine residues by catalysing the reduction of methionine sulfoxide back to methionine, thereby restoring protein function and stability [27]. Metal chelation is another mechanism of action of some antioxidants. Metal ions such as Cu^2+^ and Fe^2+^ can catalyse the generation of reactive hydroxy species, which damage cells. Antioxidants, such as flavonoids (polyphenols), can chelate or bind these metal ions, inhibiting the generation of these free radicals and limiting cellular damage [28]. Other mechanisms, such as the quenching of singlet oxygen formation by carotenoids to prevent ROS activity and antioxidant regeneration to maintain a protective pool, are known [25,29] (Figure 1).

Singlet oxygen molecules are highly reactive excited states of oxygen that can cause oxidative stress in cells. Singlet oxygen quenching by some antioxidants neutralises oxidative activities, and this can occur via physical quenching or absorption of excitation energy by antioxidants (non-reactive energy transfer), such as carotenoids [30]. It can also occur through chemical quenching, which is when the excited molecules react with antioxidants such as phenols, ascorbic acids, and polyphenols to form non-oxidative compounds [31,32]. Antioxidant regeneration refers to when a primary antioxidant, which is in a reduced state/activity (oxidised state) after neutralising the reactive species, is restored to its functional state and original activity by a secondary antioxidant [33].

## 3. Importance of Antioxidants

Antioxidants have many benefits; however, for this review, the focus will be on their health and industrial benefits using the food industry as a case study.

Antioxidants, whether endogenous or exogenous, can benefit human health. This is because they constitute a line of mutually supportive defence necessary for maintaining the oxidant/antioxidant balance in the body, with associated health benefits [34]. Some natural antioxidants may enhance the efficacy of cancer therapy and reduce toxicity [35] and bladder cancer risks [36]. Reductions in blood pressure, enhanced insulin sensitivity, improvement in eye sight, skin disease treatments, and cardiovascular outcomes in trials involving the application of different antioxidants such as lycopene, astaxanthin, lutein, β-carotenes, and canthaxanthin are known [18,37,38,39]. Unfortunately, human beings are unable to synthesise these carotenoids; therefore, they are diet-derived [40,41]. A review of the literature by [42] has shown that plant-sourced phenolic compounds, such as anthocyanins, flavonols, flavones, and phenolic acids, found in dairy products (such as milk and cheese), animal feeds, or added as additives, enhance their nutritional profile and confer multiple health benefits, including antidiabetic, cardioprotective, neuroprotective, and anticancer properties. There are excellent reviews on the use of antioxidants as nutraceuticals and pharmaceuticals [43,44].

Antioxidants are beneficial to the food industry, where they can be used to extend the shelf-life of food products via inhibition of adverse oxidative processes or their potential incorporation in food packaging in active packaging and to maintain and improve the sensory, nutritional, and health profiles of these products. For example, natural antioxidants such as olive biphenols can be used to extend the shelf lives of beef, pork, and fish products by reducing the rate of lipid oxidation (up to 83%), preserving vitamin and omega-3 levels, thereby maintaining the organoleptic properties of these foods [45]. Similarly, terpenoids (monoterpenes, carotenoids), polyphenols (phenolic acids, flavonoids, lignans), lignin/nanolignins from plants have been shown in various studies to preserve and/or extend the shelf life of meat and fish products when added as additives or into the packaging matrix, as in the case of lignin [46,47,48]. Other reviews have shown that the antimicrobial and antioxidant properties of the phenolic components of essential oils have been utilised to maintain the quality and shelf lives of meat, fruits, and vegetables [49].

Other benefits of antioxidants include their use in creams and topical application to protect the skin from oxidative and UV damage (anti-ageing, photoprotection) [50]. They have also been used as nutritional supplements to improve the yield (weight gain) of fish in aquaculture [51,52]. Antioxidants have also been used in the textile industry and as stabilised enzymes in research activities [53].

## 4. Sources of Natural Antioxidants

Given the challenges associated with synthetic antioxidants, naturally occurring antioxidants with lower toxicity are preferable, and this is the focus of this review. Natural antioxidants can be a natural component of the living system or introduced into the system via diet, usually through food and supplements, with these antioxidants invariably derived from living systems such as plants, animals, and microorganisms [4]. Natural antioxidants have been obtained from or demonstrated in bacteria [54], fungi [14], fruits, vegetables, and plants in general [55]. While comprehensive reviews of antioxidants with respect to their sources, types, mechanisms, and applications from natural and synthetic sources abound [1,4,25], limited information on these antioxidants from a special group of biological systems, extremophilic systems, is available.

## 5. Extremophiles as a Source of Antioxidants

Extremophilic biological systems (microorganisms) are a rich source of stable antioxidants due to their evolutionary adaptation to surviving in extreme conditions, such as those of temperature, acidity, and salinity, compared to those from conventional biological systems [18,56,57]. This is a key advantage as extremophiles produce structurally stable and robust antioxidants (enzymes) that can maintain their functionality under conditions that would have adversely affected antioxidants from other sources. Additionally, some of these antioxidants are unique and often have superior catalytic activities compared to antioxidants from other sources [20]. Therefore, high-value robust and potent antioxidants such as phenolics, specialised enzymes, carotenoids, and rare biomolecules with elevated oxidative capacities, are prime candidates for industrial exploitation [18,19,58,59,60]. Consequently, they are potentially suitable for different industrial and biotechnological applications in the food, cosmetic, and pharmaceutical sectors. Using enzymes from extremophilic organisms for industrial purposes offers important operational benefits. The ability of extremophiles to grow under extreme environmental conditions, such as extremes of temperature, salinity, and pH (inhibitory to most microorganisms), helps minimise contamination risks by other microbial groups. This reduces operational costs, improves process efficiency and stability, particularly when employed in large-scale industrial cultivation and production systems [18,19]. Therefore, this intrinsic stability under harsh conditions should offer a clear competitive advantage over conventional biological sources. Therefore, extremophilic systems are expected to be able to sustain the required efficiency for antioxidant biosynthesis and yield improvement during industrial operations. Consequently, antioxidants derived from extremophilic biological systems should attract significant scientific and industrial interest and, when available, could be preferred to those derived from conventional sources, especially if stress-adapted antioxidant generation pathways are optimised for greater yield. Some of the different types of extremophiles, such as acidophiles, alkaliphiles, barophiles, psychrophiles, halophiles, and radiophiles, are known (Table 1) [61,62].

### 5.1. Sources of Extremophilic Antioxidants: Microalgae

Microalgae are a diverse group composed of photosynthetic prokaryotic and eukaryotic microorganisms, rich in lipid and other biomolecules, which are found in aquatic and terrestrial environments [73,74,75]. As photosynthetic microorganisms, they convert water, carbon dioxide, and inorganic salts into biomass by using sunlight as an energy source [17]. Extremophilic microalgae are characterised by their ability to grow under extremely acidic or alkaline pH, very high and very low temperatures, and extremes of CO_2_, salts, and metal concentrations [76,77,78].

Extremophilic microalgae are excellent sources of various robust antioxidants, including SODs, carotenoids, and other antioxidant systems. Extremophilic red microalga *Cyanidioschyzon merolae* (Rhodophyta) from sulfuric hot springs is both a thermophile, growing at temperatures of up to 56 °C, and an acidophile that is nickel resistant under laboratory conditions [78,79,80]. The adaptive laboratory evolved microalga has a lower amount of ROS compared to the wild type due to the high activity of its Ni-SOD [78]. Sulphur starvation of the extremophilic microalgae *Galdieria sulphuraria* (Rhodophyta) has been shown to induce SOD as an antioxidant to combat nutrient stress [81]. SOD levels were observed to dramatically increase in snow algae *Chloromonas nivalis* (Chlorophyta) when stressed at 4 °C under laboratory conditions, suggesting that this antioxidant system contributed to ROS removal in this organism [82]. Genes coding for SODs have also been observed in halo-tolerant *Dunaliella salina* (Chlorophyta) (a well-known carotenoid-producing algae) under low salinity [83].

Acidophilic algae, such as *Coccomyxa onubensis* (Chlorophyta), have been shown to tolerate high heavy metal concentrations/stress (Arsenic, cadmium, mercury, and copper) through three different antioxidant mechanisms: the ascorbate peroxidase (APX), catalase (CAT), and glutathione reductase systems [84]. Under stressful conditions, most extremophilic microalgae use multiple antioxidative systems. For example, in snow algae, *Chloromonas nivalis and* other microalgae such as *Dunaliella salina* and *Galdieria sulphuraria* are also known to use the catalase system alongside the SOD [82].

Carotenoids and pigments are antioxidants that are produced by extremophilic microalgae in laboratory-based assays and in the natural environment. Thermoacidophilic red alga *Galdieria sulphuraria* found in acidic hot springs (can grow at 56 °C and at low pH) [85] is known to produce a phycocyanin, with proven antioxidant activities through HAT and SET, with the pigment used as a natural colourant in the food industry [24]. *Sanguina aurantia* and *Sanguina nivaloides* (Chlorophyta) found in arctic and subarctic regions produce astaxanthin [86]. *Chromochloris zofingiensis* also synthesises carotenoids such as canthaxanthin and astaxanthin [87]. *Coccomyxa melkonianii* SCCA 048 (heavy metal resistant) is known to synthesise significant amounts of lutein (up to 80% of total carotenoid) [88] while acidophilic *Coccomyxa onubensis* and *Chlamydomonas acidophila* can produce β-carotene and lutein [89,90,91]. *Deinococcus xibeiensis* R13 (radiation- and UV-resistant extremophile) produces Deinoxanthin-type carotenoids (xanthophylls) in significant amounts [92].

Literature reviews have shown that other extremophilic groups, such as *Dunaliella salina* (β-carotene production), *Arthrospira platensis* (c-phycocyanin), *Tetradesmus dissociates* (Chlorophyta) (canthaxanthin production), and *Haematococcus lacustris* (formerly *Haematococcus pluvialis*) (Chlorophyta) (astaxanthin production), all have beneficial antioxidants [19,93]. A review of the literature by [18] has shown that extremophilic *Tetradesmus dissociates* MT1 (from the Sahara desert) and halophilic/tolerant *Chromochloris zofingiensis* (Chlorophyta) use canthaxanthin as their preferred antioxidant system. Psychrophilic microalgae such as *Scenedesmus* sp. (Chlorophyta), *Chlorococcum* sp. (Chlorophyta), and halophilic *Dunaliella* sp. (Chlorophyta) also produce antioxidative compounds such as lutein, carotene, and antheraxanthin [94,95]. A summary of some of these examples is presented in Table 2.

### 5.2. Sources of Extremophilic Antioxidants: Bacteria and Archaea

An excellent review by [96] has identified an antioxidant system, the superoxide dismutases (SOD), from extremophilic bacteria, which are more stable than SOD from other sources. These SOD are enzymes that can convert toxic superoxide radicals into hydrogen peroxide and oxygen, thus protecting bacterial cells from free radical-induced damage [2]. Enzymes such as catalase or glutathione peroxidase can further break down hydrogen peroxide into water and oxygen [2,125]. For example, *Thermus thermophilus* HB27, originally isolated from a natural hot spring in Japan, is a thermophilic and halophilic bacterium that expresses its antioxidative properties via a robust superoxide dismutase (SOD) that is highly stable at 90 °C [96,97]. Manganese-dependent SODs (active at up to 80 °C and a pH range of 3.0–12.0) have been observed in extremophilic *Bacillus subtilis* (Firmicutes) and *Thermus thermophilus* (Deinococcota) [2].

Other SODs have been reported in the extremophilic *Acinetobacter* sp. (Proteobacteria) [126] and thermoacidophilic *Alicyclobacillus* sp. (Firmicutes) [127]. Conversely, cold-adapted SODs are also known; a good example is the Cu/Zn SOD found in a recombinant strain, *Halomonas* sp. (Proteobacteria) ANT108, derived from the extremophilic sea-ice strain *Halomonas* sp. [98]. However, the optimum temperature for SOD activity was 35 °C, with 13.9% activity at 0 °C [98]. Fe/Mn SOD has been characterised in *Bacillus licheniformis* (Firmicutes) [128] and Fe SOD in *Thermobifida fusca* (Actinobacteria) [129].

Another antioxidant system encountered in extremophilic bacteria is the catalase system [96]. Excess hydrogen peroxide and hydroxyl radicals can be harmful to cells, with this damage mitigated by catalases, which, as previously stated, can convert these compounds into water and oxygen. A cold-adapted heme catalase system, KatE1, has been identified in an extremophilic *Acinetobacter* (Proteobacteria)isolate from the Atacama Desert plateau (characterised by high UV radiation, salinity, and extremes of temperature) in Argentina [99]. This catalase system was active at up to 50 °C (maximum activity) in laboratory-based assays with “nearly unchanged specific activities” from pH 5.5 to 11 and 0 °C to 40 °C [99]. Similarly, a psychrophilic *Serratia* sp. (Proteobacteria) with a highly stable and thermoactive UV-C-resistant catalase system has been isolated from Elephant Island, in Antarctica [100]. Remarkably, this catalase was active between 20 and 70 °C, with optimal activity at 50 °C and pH 7.0 [100]. Reviews of the literature by [96] have identified different catalase systems, such as a thermophilic Mn-catalase (active at 75 °C and a pH of 9.0) from *Geobacillus* sp. (Firmicutes) strain WCH70, a thermoalkali-stable heme catalase (active at 90 °C) from *Thermus brockianus* (Deinococcota), and a psychrophilic catalase system (0 and 10 °C) from *Aliivibrio salmonicida* (Proteobacteria). Hyperthermophilic archaea such as *Methanosarcina barkeri, Methanobrevibacter arboriphilus,* and *Halobacterium halobiuare* (Euryarchaeota/halobacteriota) are also known to produce catalases [15].

Additionally, carotenoids with higher antioxidant activity than those from conventional sources, such as blueberries and carrots, have been reported in halophilic groups. Laboratory-based assay of carotenoids from *Halobacterium salinarum* (an archaeon) showed that antioxidant activities from this extremophile were almost 2-fold higher than those of carotenoids from berries [101]. Carotenoids have also been obtained from an extremophilic *Arthrobacter* sp. (Actinomycota) [103]. Similarly, halophilic *Haloterrigena turkmenica* (Euryarchaeota), an extremophilic archaeon, is known to mainly secrete C50 carotenoids to protect it from oxidative species in laboratory-based assays [102]. In general, hyperthermophilic archaea use a variety of catalases, SOD, and peroxiredoxins to scavenge destructive ROS in the environment [15]. Furthermore, radioresistant Deinococcus radiodurans (Deinococcota) is well known for its utilisation of antioxidant proteins, low molecular weight manganese complexes, and carotenoids (deionoxanthin) [20,103,104,105] and a Cu/Zn SOD system [106] to protect it from ionising radiation and to repair radiation-induced DNA damage. Similarly, extremophilic halophiles, *Halococcus morrhuae* (Euryarchaeota), *Halobacterium salinarium* (Euryarchaeota), and *the bacterium, Thermus filiformis* (Deinococcota), are also known to produce carotenoid groups [130].

Cyanobacteria are another bacterial group that is known to produce antioxidants. Extremophilic cyanobacteria can be classified as psychrophiles, alkaliphiles, thermophiles, xerophiles, and halophiles based on their habitat [131]. These groups can produce potent and robust UV-absorbing antioxidant compounds such as scytonemin and mycosporine-like amino acids alongside other commonly known antioxidants like SODs, catalase, and carotenoids as adaptations to their harsh growth environments. Scytonemin (pigments) and mycosporine-like amino acids (MAAs) are produced by cyanobacteria to protect themselves from the oxidative stress associated with ultraviolet (UV) radiation, enabling them to survive in different environments, including those characterised by UV radiation, temperature extremes, and low water activity [107].

Cyanobacterium *Nostoc* (Cyanobacteriota) commune Vaucher ex Bornet et Flahault, isolated from Antarctica, has been shown to produce substantial levels of scytonemin for UV-associated oxidative stress reduction [108]. Others, such as *Leptolyngbya* cf. *fragilis* (Cyanobacteriota), were isolated from a hot spring [109], endolithic *Halothece* (Cyanobacteriota) cyanobacteria from the Atacama Desert [110] and *Chroococcidiopsis* sp. (Cyanobacteriota). UAM571 [111] also isolated from the same desert were found to produce increasing levels of scytonemin under stress. Substantial levels of UV-protecting mycosporine-like amino acid (MAA) and carotenoids had been identified in *Phormidium* sp. (Cyanobacteriota) isolated from Antarctica [112]. *Aphanothece halophytica* (Cyanobacteriota), a halotolerant cyanobacterium that produces mycosporine-2-glycine, has been isolated from Solar Lake in Sinai [19]. A summary of some of these examples is presented in Table 2.

### 5.3. Sources of Extremophilic Antioxidants: Fungi

Like other reviewed biological systems, extremophilic fungi utilise standard antioxidative systems, which can be enzymatic (e.g., superoxide dismutases, peroxiredoxins, and catalases) or non-enzymatic (e.g., carotenoids and melanin). These systems scavenge ROS and neutralise free radicals. As reported for microalgae and bacteria, these systems enable them to survive habitat-related stressors, such as extremes of temperature, either very hot, like in the desert, or very cold, as in Antarctica, extremes of pH, low water activities/drought, metal toxicity, high salinity, and high radiation [132]. A brief review of reported examples of extremophilic fungi and their antioxidant systems is presented in the following paragraphs, with relevant references.

Catalase systems have been identified in Antarctic *Aspergillus fumigatus* (Ascomycota) [113], Penicillium rubens [114], and *Aspergillus glaucus* (Ascomycota), with the latter having a functional ROS-scavenging SOD [115]. A comprehensive review of other enzymatic antioxidant systems, such as peroxiredoxins, thioredoxins, and glyoxalases, has been carried out [133].

Melanin, a pigment, is known to be one mechanism by which some extremophilic fungi protect themselves from oxidative damage [120]. Different types of melanin have been identified in extremophilic fungi. These include DHN melanin (1,8-Dihydroxynaphthalene) (allomelanin), pyomelanin, and L-DOPA-melanin (L 3,4-dihydroxyphenylalanine) (Eumelanin/pheomelanin), with DHN and L-DOPA melanin being commonly associated with fungi belonging to the basidiomycetes and the ascomycetes [134,135]. Both eumelanin and allomelanin exhibit strong antioxidant properties via well-studied mechanisms. Both can scavenge free radicals such as DPPH (1,1-diphenyl-2-picrylhydrazyl) and ABTS (2,2′-azino-bis(3-ethylbenzothiazoline-6-sulfonic acid), which are involved in ROS and UV radiation and redox cycling [136,137,138,139,140]. While there is sometimes metabolic flexibility with both pathways existing in some fungal genera, such as *Cryomyces antarcticus* [116], it appears that, in general, the basidiomycetes primarily produce eumelanin using tyrosine-dependent pathways, while ascomycetes largely synthesise allomelanin using a different pathway, such as the polyketide pathway [141,142].

Antarctic black fungus *Cryomyces antarcticus* (Ascomycota) is an example of a fungus that is known to produce this pigment, which protects it from the oxidative effects of temperature stress, UV radiation, and desiccation [116,117]. *Exophiala dermatitidis*, a polyextremotolerant yeast, is also known to synthesise DHN melanin [118]. Melanin is also known to protect black yeasts, which are commonly found in hypersaline conditions [121,143].

Carotenoids have also been reported in cold-adapted *Knufia petricola* A95 [119] and in other extremophilic fungi such as Dioszegia patagonica (Basidiomycota) and Antarctic *Arthrobotrys* sp. (Ascomycota) [120,122,123] and in some halophilic *Fusarium* sp. (Ascomycota) [121,124]. Other compounds, such as phenols, xanthones, anthraquinones, and alkaloids, which scavenge ROS, have been reported in Antarctic fungi [9]. A summary of some of these examples is presented in Table 2.

## 6. Applications of Antioxidants from Extremophilic Microorganisms

Antioxidants such as superoxide dismutases (SODs), catalases, and carotenoids, obtained from extremophilic biological systems, have various applications owing to their stability and their ability to combat oxidative stress. Some of these applications (current and potential) are presented in the following paragraphs and summarised in Table 3.

### 6.1. Superoxide Dismutases (SODs)

Superoxide dismutases are antioxidant enzymes found in most living systems, which are involved in the breakdown of superoxide radicals into hydrogen peroxide and molecular oxygen. The hydrogen peroxide is later converted into water and oxygen by enzymes such as peroxidases and catalases [144,145]. SODs with high catalytic functions have been identified in extremophilic organisms, and given their stability under varying conditions, they are an excellent source of biocatalysts for industrial applications [145]. A review of SOD, its different forms, such as Cu-Zn SOD, FE-SOD, Mn-SOD, and Ni-SOD, has been carried out [144].

SODs have potential applications in the medical industry as they can reduce and/or prevent oxidative stress associated with a variety of illnesses such as cardiovascular diseases, cancer, diabetes, infertility, neurological disorders, rheumatoid arthritis, and asthma. Their stability at high temperatures (up to 80 °C) and extreme pHs makes them excellent candidates for potential therapeutic applications for these different diseases [2]. These SODs can potentially be incorporated or engineered into different pharmaceutical products (e.g., antioxidant creams and medications) to provide robust antioxidant protection. For example, Mn-SOD from thermoacidophilic *Alicyclobacillus* (Firmicutes), which is stable at temperatures up to 80 °C and a pH range of 2–10 (stable in acidic stomach-like environment), has been touted as a candidate for use in medical applications and as a food additive because of its inherent robustness [127].

They can also be used in cosmetics and skin care products as biosensors and for product preservation. For example, SODs from the thermo-halophilic *Aeropyrum pernix* (Thermoproteota) (SODAp) and the thermo-acidophilic *Saccharolobus solfataricus* (Thermoproteota) (SODSs) heterologously expressed in transgenic tomato cell cultures using recombinant DNA technology were stable at high salt, low pH, and high temperatures. Therefore, they can be used as natural additives for food preservation or as dietary supplements [145]. Extracted SODs from thermophilic organisms *Oceanobacillus saliphilus* (Firmicutes) and *Geobacillus thermoleovorans* (Firmicutes) were found to show significant resistance to heat and extreme pH conditions, compared to traditional mesophilic SODs, indicating that they will be excellent candidates for use in cosmetic, medical, and industrial applications [2].

Some current commercial uses of SOD are briefly discussed. The review by [96] identified SODs from extremophiles, or those engineered to be stable at high temperatures. These include Biocell^TM^ SOD (from Lonza (Singapore), stable at 45 °C, used in skin and hair care products) and Native *Geobacillus stearothermophilus* (firmicutes) SOD (from Creative Enzymes (Shirley, NY, USA), active at 60 °C, with medical, cosmetic, and nutritional applications). However, despite their enormous potential, evidence of their use is limited because significant challenges remain in translating this potential into medical products with reliable delivery systems and sustained product efficiency to achieve desired product outcomes.

### 6.2. Catalases

Catalases from extremophilic organisms can potentially be used in different industries, including those involved in biomedicals, food, manufacturing (e.g., the textile industry), and environmental bioremediation [146,147]. They are used as biocatalysts due to their stability and robust performance under extreme conditions [96,148]. This gives them a competitive advantage over catalases from mesophilic systems, which may struggle to function optimally under extremes of environmental conditions (such as extreme temperatures, pH levels, and salinity) and stressful industrial conditions [148].

Catalases from extremophilic and non-extremophilic sources can be used to remove hydrogen peroxide from fabrics in the textile industry [149]. Current applications of such catalases for industrial use include the brand Oxy-Gone^®^ T400 (active at 30–70 °C), manufactured by Genencor (Palo Alto, CA, USA), which is used to remove hydrogen peroxide from textiles before textile dye application [96]. Similarly, Conzyme^®^ CAT50 (Sunsozymes, Beijing, China) and Terminox (Novozymes, Kalundborg, Denmark), both active at up to 50 °C, are used to remove residual hydrogen peroxide after textile bleaching as part of post-bleaching clean-up processes before other downstream processes [96].

These catalases are also used in hydrogen peroxide removal in the food industry, for research, diagnostics, and wastewater treatment [150]. For example, Swissaustral commercially produces and sells thermostable catalase in both liquid and powder forms for research and industrial applications (https://www.swissaustral.com/shop/category/1, accessed on 18 February 2026). Another company, Creative Enzymes, sells a thermostable fungal catalase used to remove hydrogen peroxide in the textile industry, semiconductor, and HPPO factories. The enzyme is stable at 70 °C (https://www.creative-enzymes.com/product/native-thermostable-fungi-catalase-for-semiconductor-process_16150.html, accessed on 18 February 2026). While catalases from non-extremophiles can be used in skincare products to neutralise free radicals and protect skin from UV radiation [151], no information on the commercial application for these enzymes from extremophiles was available.

### 6.3. Carotenoids

The carotenoids market is growing and is estimated to reach USD 2.0 billion by the year 2026, with the most valuable examples of marketed carotenoids including astaxanthin, canthaxanthin, β-carotene, lutein, and lycopene [152]. They have widespread application in the cosmetic industry, the aquaculture industry, animal husbandry, and in the food industry as food colourants, food and feed additives, and supplements [153]. In the medical industry, reviews of the literature have shown that these carotenoids are anti-inflammatory, anticancer, improve cardiovascular health (astaxanthin, lutein, and lycopene), prevent liver fibrosis, and prevent night blindness (β-carotene) [152]. A review of the therapeutic uses of natural astaxanthin based on evidence from human clinical trials has been carried out [37].

Microalgae are excellent feedstock for high-value compounds. For example, *H. lacustris (pluvialis)* is one of the most commercially important microalgae because it is the primary source of the carotenoid, astaxanthin, which is considered to be a “super antioxidant” because of its high potency, as its antioxidant activities are ten times higher than those observed in lutein or β-carotene [18,154]. *H. lacustris* (*pluvialis*) is known to naturally produce the highest concentration of astaxanthin (5% of antioxidants on a dry weight basis) under stressful conditions [18,19]. It is a high-value microalgal product that has already been commercialised as a nutritional and health supplement in foods and in animal feeds. Cryophilic microalga *C. nivalis* is also known to produce significant levels of astaxanthin [19].

Astaxanthin from *H. lacustris* (*pluvialis*) has been marketed in various formulations for food use as dietary supplements after regulatory approval in countries such as the US and UK. Major producers include DSM (Maastricht, The Netherlands), BASF (Levallois-Perret, France), NHU (Shaoxing, China), Cyanotech (Kailua-Kona, HI, USA), AstaReal (Nacka, Sweden), and Algatech (Harrison, NY, USA) [155,156]. Astaxanthin-based products are available in different forms: syrups, tablets, soft gels, granulated powders, oils, and creams. Other forms of regulatory approval have been secured in multiple jurisdictions: *H. lacustris* (*pluvialis*) CO_2_-based extracts were granted a “novel food” status by the UK Food Standards Agency (FSA). In contrast, astaxanthin from *H. lacustris* (*pluvialis*) was given “GRAS” status (Generally Recognised As Safe) by the US FDA (Food and Drug Administration) [157,158].

β-carotene is commercially produced from *Dunaliella salina*, which can tolerate highly saline conditions exceeding 300 g/L [18]. The latter *Dunaliella salina* can accumulate up to 14% β-carotene by dry weight, far more than any synthetic variants in antioxidant activity [19,159]. Canthaxanthin is a lipid-soluble antioxidant derived from the halophilic archaeon *Haloferax alexandrine* (Euryarchaeota). It is used as a feed additive in agriculture, in poultry, fish, and crustacean farms, and is also used in the cosmetic industry as the key ingredient in tanning pills [160,161]. Detailed reviews of the sources and the commercial applications of other carotenoids, such as canthaxanthin and lutein (xanthophylls), have been carried out [19]. In contrast, carotenoids from extremophilic microbial groups such as *Halococcus morrhuae* (archaea) and *Thermus filiformis* (bacteria), despite their potent antioxidant properties, are yet to be successfully commercialised. Deinoxanthin (DXT), a C40 monocyclic carotenoid produced by the extremophilic *Deinococcus radiodurans*, which is a promising food-grade antioxidant pigment and a potential anti-tumour agent, has yet to find a commercial application [20,103].

## 7. Conclusions

This review highlights the potentially substantial functional advantages of extremophilic antioxidants compared with those from conventional sources. Their intrinsic stability under extreme conditions, including temperature, salinity, and pH, makes them excellent candidates for biotechnological, industrial, and biomedical applications. Using specific examples such as superoxide dismutase, catalase, carotenoids, melanin, and peroxidases, their outstanding abilities to survive and maintain high operational efficiency under harsh, stressful conditions that would inactivate conventional enzymes are discussed. These underscore their potential for use in medicine (pharmaceuticals), food (nutritional supplementation and preservation), agriculture, cosmetics, and other industries. Overall, extremophile-derived antioxidants are an emerging, stable, and potent resource for combating oxidative stress and for nutritional supplementation to improve health outcomes, enhance product stability and preservation, and support research and sustainable bioprocessing. However, challenges remain in developing cost-effective production and delivery systems that are not adversely affected in functionality, highly scalable, and capable of overcoming regulatory hurdles.

Future research should focus on obtaining more extremophilic microorganisms from other extreme environments, such as caves, and on expanding genomic and molecular exploration of understudied extremophiles to identify novel antioxidant enzymes with desirable properties. For most extremozymes, gaps currently exist between laboratory-validated stability and functionality and their real-life applications, underscoring the need for large-scale bioprocess optimisation, bioreactor design, and downstream purification. This is critical to making their commercial production economically viable. Structural biology studies, molecular engineering, increased use of recombinant approaches for therapeutic and industrial applications, integration of artificial intelligence, multi-omics, machine learning, and synthetic biology will accelerate the discovery and development of new extremophilic antioxidants. This will position extremophilic antioxidants as next-generation tools for biotechnology and medicine.

## Figures and Tables

**Table 1:** The Different Types of Extremophilic Microorganisms.

Designation	Extreme Conditions	Habitat and Examples
Acidophiles	Acid-loving. Grows at a pH below 5. Optimum pH is 3.	Hydrothermal vents, mine sites, and geothermal acidic sites. Examples include *Acidithiobacillus thiooxidans* and *Acidithiobacillus thiooxidans* [61,63].
Alkaliphiles	Alkali-loving. Grows at pH 9 and above.	Manufacturing effluent, mining operations, paper and pulp production, and alkali lakes. Examples include *Bacillus* sp., *Acremonium*, and *Sodiomyces* species [61,64,65]
Capnophiles	Grows at a CO_2_ concentration of 1–10%.	Clinical samples (urine of patients with severe pyelonephritis) and cold-water geysers. Examples include *Lactobacillus*, *Proteus mirabilis*, *Streptococcus pneumonia,* and *E. coli* [61,66]
Halophiles	Salt-loving. Grows at 1.5–4.0 M salt concentrations	Salt lakes, marshes, and ponds, and manufacturing effluents. Examples include *Haloquadratum walsbyi*, *Dunaliella* sp., and *Actinarchaeum halophilum* [61,67]
Metallophiles	High metal concentration-loving	Manufacturing effluent, mine tailings, mine sites, contaminated groundwater, and aquifers. Examples include *Acinetobacter*, *Citrobacter*, *Pseudomonas,* and *Thiobacillus* [61,68]
Piezophiles	Pressure-loving, up to 80 MPa	Deep-sea environments, underground water. Examples include *Salinimonas sediminis*, *Colwellia marinimaniae,* and *Shewanella benthica* [61,69]
Psychrophiles	Cold-loving, grows from −20° to 20 °C	Arctic and Antarctica, permafrost, deep-sea, and cold storage facilities. Examples include *Alcaligenes*, *Achromobacterium*, *Alteromonas,* and *Colwellia* sp. [61,68]
Radiophiles	Radiation-loving, 1500–6000 Gy	Nuclear waste, ionised products. Examples include Deinococcus radiodurans [61,70]
Thermophiles	High temperature-loving, grows from 46 °C to >75 °C	Hot springs, geysers, manufacturing effluents, and deserts. Examples include *Thermus*, *Bacillus,* and *Geobacillus* sp. [61,71]
Xerophiles	Dry habitat-loving	Deserts and any other dry environments. Examples include *Aspergillus penicillioides* and *Xeromyces bisporus* [61,72]

Note: Adapted from [61].

**Table 2:** Diversity of extremophilic microorganisms and their antioxidant systems.

Organism (Group)	Extremophilic Trait	Major Antioxidants	Functional Role/Mechanism	References
*Cyanidioschyzon merolae* (Rhodophyta)	Thermoacidophile; Ni-resistant	Ni-SOD	Reduced ROS via enhanced SOD activity in evolved strains	[78,79,80]
*Galdieria sulphuraria* (Rhodophyta)	Thermoacidophile; nutrient stress-tolerant	SOD, catalase, phycocyanin	SOD induced under sulphur starvation; ROS detoxification; pigment antioxidant	[81,85]
*Chloromonas nivalis* (Chlorophyta)	Psychrophile	SOD, catalase	Increased SOD activity at low temperature for ROS removal	[82]
*Dunaliella salina* (Chlorophyta)	Halotolerant	SOD, β-carotene	SOD gene expression; carotenoid antioxidant system	[19,83,93]
*Coccomyxa onubensis* (Chlorophyta)	Acidophile; heavy metal tolerant	APX, CAT, glutathione reductase, lutein	Detoxification via multiple antioxidant pathways	[84,89,90,91]
*Chromochloris zofingiensis* (Chlorophyta)	Halotolerant	Canthaxanthin, astaxanthin	Carotenoid-mediated ROS scavenging	[18,87]
*Sanguina aurantia*/*S. nivaloides*	Psychrophile	Astaxanthin	UV and oxidative stress protection	[86]
*Coccomyxa melkonianii*	Heavy metal resistant	Lutein	High carotenoid accumulation	[88]
*Thermus thermophilus*	Thermophile/halophile	SOD, catalase	Thermostable antioxidant enzymes	[96,97]
*Halomonas* sp. ANT108	Psychrophile	Cu/Zn SOD	Cold-active antioxidant enzyme	[98]
*Acinetobacter* sp.	Cold/desert extremophile	Catalase (KatE1)	Broad pH and temperature activity	[99]
*Serratia* sp.	Psychrophile	Catalase	UV-C-resistant catalase	[100]
*Halobacterium salinarum*	Halophile	C50 carotenoids	Strong antioxidant activity	[101]
Haloterrigena turkmenica	Halophile	C50 carotenoids	Oxidative stress protection	[102]
*Deinococcus radiodurans*	Radioresistant	Deinoxanthin, SOD, Mn-complexes	Radiation protection and DNA repair	[20,103,104,105,106]
Cyanobacteria (e.g., *Nostoc*, *Chroococcidiopsis*)	UV tolerant	Scytonemin, MAAs, carotenoids	UV absorption and ROS scavenging	[107,108,109,110,111]
*Phormidium* sp.	Psychrophile	MAAs, carotenoids	UV protection	[112]
*Aspergillus fumigatus*/*Penicillium rubens*	Antarctic fungi	Catalase	ROS detoxification	[113,114]
*Aspergillus glaucus*	Extremophilic fungus	SOD, catalase	ROS scavenging	[115]
*Cryomyces antarcticus*	Polyextremophile	DHN-melanin	Protection against UV, ROS, and desiccation	[116,117]
*Exophiala dermatitidis*	Polyextremotolerant yeast	DHN-melanin	Stress protection	[118]
*Knufia petricola*	Cold-adapted fungus	Carotenoids	ROS scavenging	[119]
*Dioszegia patagonica*/*Fusarium* sp.	Cold/halophilic fungi	Carotenoids	Oxidative stress protection	[120,121,122,123,124]

**Table 3:** Commercial antioxidant products derived from extremophiles.

Product/Compound	Commercial Product Name	Manufacturer	Source Organism	Extremophile Type
β-carotene	Betatene^®^	BASF (Fargo, ND, USA)	*Dunaliella salina*	Halophilic microalga
Astaxanthin	BioAstin^®^	Cyanotech Corporation (Kailua-Kona, HI, USA)	*Haematococcus pluvialis*	Stress-tolerant microalga
Phycocyanin	LinaBlue^®^	DIC Corporation (Irvine, CA, USA)	*Arthrospira platensis* (Spirulina)	Alkaliphilic cyanobacteria
Mycosporine-like amino acids (MAAs)	Helioguard^®^ 365	Mibelle Biochemistry (Buchs, Switzerland)	Red algae/cyanobacteria	UV-resistant extremophiles
Ectoine	Ectoin^®^	Bitop AG (Dortmund, Germany)	*Halomonas elongata*	Halophilic bacteria
Exopolysaccharides (EPS)	Abyssine^®^	Lucas Meyer Cosmet-ics (Québec, QC, Canada)	*Alteromonas macleodii*	Piezophilic (deep-sea bacteria)

## Data Availability

No new data were created or analyzed in this study. Data sharing is not applicable to this article.

## References

[1] Gulcin İ. (2025). Antioxidants: A Comprehensive Review. Arch. Toxicol..

[2] Zhang N., Ren Z., Wei D., Yang M., Niu M., Shen C., Jin X., Wei M., Choi J., Park M. (2025). Discovery, Expression, and Characterization of Highly Tolerant Superoxide Dismutases from Extremophiles for Potential Industrial Applications. Int. J. Biol. Macromol..

[3] Flieger J., Flieger W., Baj J., Maciejewski R. (2021). Antioxidants: Classification, Natural Sources, Activity/Capacity Measurements, and Usefulness for the Synthesis of Nanoparticles. Materials.

[4] Halliwell B. (2024). Understanding Mechanisms of Antioxidant Action in Health and Disease. Nat. Rev. Mol. Cell Biol..

[5] Pan M., Liu K., Yang J., Liu S., Wang S., Wang S. (2020). Advances on Food-Derived Peptidic Antioxidants—A Review. Antioxidants.

[6] Clemente-Suárez V.J., Bustamante-Sanchez Á., Mielgo-Ayuso J., Martínez-Guardado I., Martín-Rodríguez A., Tornero-Aguilera J.F. (2023). Antioxidants and Sports Performance. Nutrients.

[7] Tauchen J., Huml L., Jurášek M., Regenstein J.M., Ozogul F. (2025). Synthetic and Semi-Synthetic Antioxidants in Medicine and Food Industry: A Review. Front. Pharmacol..

[8] Pisoschi A.M., Pop A. (2015). The Role of Antioxidants in the Chemistry of Oxidative Stress: A Review. Eur. J. Med. Chem..

[9] Abrashev R., Miteva-Staleva J., Gocheva Y., Stoyancheva G., Dishliyska V., Spasova B., Krumova E., Angelova M. (2025). Cell Response to Oxidative Stress in Antarctic Filamentous Fungi. Appl. Sci..

[10] López-Pedrouso M., Lorenzo J.M., Franco D. (2022). Advances in Natural Antioxidants for Food Improvement. Antioxidants.

[11] Shoker R.M.H., Al-Shammery W.H., Al-Aidy S.R. (2023). A Review Article: Free Radical and Replacement Synthetic Antioxidant by Natural Antioxidant. J. Res. Appl. Sci. Biotechnol..

[12] Ebrahimirad F., Mirmahdizade S.E., Mahmoodieh B., Najafi S., Banihashemian S.M., Nikakhtar S., Mobaraki H., Sadeghi A., Kossari N., SadatRafiei S.K. (2025). Antioxidant Strategies against Cellular Senescence: Unveiling the Power of Synthetic versus Natural Antioxidants in a Systematic Review. Front. Aging.

[13] Arslan N.P., Azad F., Orak T., Budak-Savas A., Ortucu S., Dawar P., Baltaci M.O., Ozkan H., Esim N., Taskin M. (2025). A Review on Bacteria-Derived Antioxidant Metabolites: Their Production, Purification, Characterization, Potential Applications, and Limitations. Arch. Pharm. Res..

[14] Arslan N.P., Dawar P., Albayrak S., Doymus M., Azad F., Esim N., Taskin M. (2025). Fungi-Derived Natural Antioxidants. Crit. Rev. Food Sci. Nutr..

[15] Pedone E., Fiorentino G., Bartolucci S., Limauro D. (2020). Enzymatic Antioxidant Signatures in Hyperthermophilic Archaea. Antioxidants.

[16] Ibrar M., Ullah M.W., Manan S., Farooq U., Rafiq M., Hasan F. (2020). Fungi from the Extremes of Life: An Untapped Treasure for Bioactive Compounds. Appl. Microbiol. Biotechnol..

[17] Malavasi V., Soru S., Cao G. (2020). Extremophile Microalgae: The Potential for Biotechnological Application. J. Phycol..

[18] Lafarga T., Sánchez-Zurano A., Morillas-España A., Acién-Fernández F.G. (2021). Extremophile Microalgae as Feedstock for High-value Carotenoids: A Review. Int. J. Food Sci. Technol..

[19] Rojas-Villalta D., Rojas-Rodríguez D., Villanueva-Ilama M., Guillén-Watson R., Murillo-Vega F., Gómez-Espinoza O., Núñez-Montero K. (2024). Exploring Extremotolerant and Extremophilic Microalgae: New Frontiers in Sustainable Biotechnological Applications. Biology.

[20] Mussagy C.U. (2025). Microbial Deinoxanthin: A Rare Carotenoid Linking Extremophiles to Functional Food Applications. Food Biosci..

[21] Maresca E., Carbone M., Gallo G., Fusco S., Aulitto M. (2025). Extremophile-Derived Bioactives in Cosmeceuticals: Bridging Nutraceuticals and Skincare for Holistic Wellness. Life.

[22] Singh L.A., Kumari P., Kumar P., Yadav A., Bhardwaj R., Swapnil P., Meena M. (2025). Microalgae-Derived Antioxidants and Antimicrobials: A Sustainable Approach for Natural Food Preservatives. Front. Sustain. Food Syst..

[23] Francenia Santos-Sánchez N., Salas-Coronado R., Villanueva-Cañongo C., Hernández-Carlos B., Shalaby E. (2019). Antioxidant Compounds and Their Antioxidant Mechanism. Antioxidants.

[24] Fitrania F., Rahman D.Y., Indrasi D., Prangdimurti E. Antioxidant Activity of Phycocyanin from *Galdieria sulphuraria* in Autotrophic Cultivation. Proceedings of the 10th ISIBIO & 13th ISISM in Conjunction with the 20th ACM Meetings.

[25] Losada-Barreiro S., Sezgin-Bayindir Z., Paiva-Martins F., Bravo-Díaz C. (2022). Biochemistry of Antioxidants: Mechanisms and Pharmaceutical Applications. Biomedicines.

[26] Jiang B., Adams Z., Moonah S., Shi H., Maupin-Furlow J., Moskovitz J. (2020). The Antioxidant Enzyme Methionine Sulfoxide Reductase A (MsrA) Interacts with Jab1/CSN5 and Regulates Its Function. Antioxidants.

[27] Chatterjee A., Sepuri N.B.V. (2024). Methionine Sulfoxide Reductase 2 Regulates Cvt Autophagic Pathway by Altering the Stability of Atg19 and Ape1 in Saccharomyces Cerevisiae. J. Biol. Chem..

[28] Sharifi-Rad M., Anil Kumar N.V., Zucca P., Varoni E.M., Dini L., Panzarini E., Rajkovic J., Tsouh Fokou P.V., Azzini E., Peluso I. (2020). Lifestyle, Oxidative Stress, and Antioxidants: Back and Forth in the Pathophysiology of Chronic Diseases. Front. Physiol..

[29] Edge R., Truscott T. (2018). Singlet Oxygen and Free Radical Reactions of Retinoids and Carotenoids—A Review. Antioxidants.

[30] Tamura H., Ishikita H. (2020). Quenching of Singlet Oxygen by Carotenoids via Ultrafast Superexchange Dynamics. J. Phys. Chem. A.

[31] Wang Y., Lin Y., He S., Wu S., Yang C. (2024). Singlet Oxygen: Properties, Generation, Detection, and Environmental Applications. J. Hazard. Mater..

[32] Niki E., Noguchi N. (2021). Antioxidant Action of Vitamin E in Vivo as Assessed from Its Reaction Products with Multiple Biological Oxidants. Free Radic. Res..

[33] Budzianowska A., Banaś K., Budzianowski J., Kikowska M. (2025). Antioxidants to Defend Healthy and Youthful Skin—Current Trends and Future Directions in Cosmetology. Appl. Sci..

[34] Janciauskiene S. (2020). The Beneficial Effects of Antioxidants in Health and Diseases. Chronic Obstr. Pulm. Dis. J. COPD Found..

[35] Boccellino M. (2023). Health Effects of Natural Antioxidants. Int. J. Mol. Sci..

[36] Tratnjek L., Jeruc J., Romih R., Zupančič D. (2021). Vitamin A and Retinoids in Bladder Cancer Chemoprevention and Treatment: A Narrative Review of Current Evidence, Challenges and Future Prospects. Int. J. Mol. Sci..

[37] Donoso A., González-Durán J., Muñoz A.A., González P.A., Agurto-Muñoz C. (2021). Therapeutic Uses of Natural Astaxanthin: An Evidence-Based Review Focused on Human Clinical Trials. Pharmacol. Res..

[38] Saini R.K., Prasad P., Lokesh V., Shang X., Shin J., Keum Y.-S., Lee J.-H. (2022). Carotenoids: Dietary Sources, Extraction, Encapsulation, Bioavailability, and Health Benefits—A Review of Recent Advancements. Antioxidants.

[39] Alugoju P., Krishna Swamy V.K.D., Anthikapalli N.V.A., Tencomnao T. (2023). Health Benefits of Astaxanthin against Age-Related Diseases of Multiple Organs: A Comprehensive Review. Crit. Rev. Food Sci. Nutr..

[40] Xavier A.A.O., Pérez-Gálvez A., Stange C. (2016). Carotenoids as a Source of Antioxidants in the Diet. Carotenoids in Nature.

[41] Rocha H., Coelho M., Gomes A., Pintado M. (2023). Carotenoids Diet: Digestion, Gut Microbiota Modulation, and Inflammatory Diseases. Nutrients.

[42] Zeb A. (2020). Concept, Mechanism, and Applications of Phenolic Antioxidants in Foods. J. Food Biochem..

[43] Anand S., Bharadvaja N. (2022). Potential Benefits of Nutraceuticals for Oxidative Stress Management. Rev. Bras. Farmacogn..

[44] Puri V., Nagpal M., Singh I., Singh M., Dhingra G.A., Huanbutta K., Dheer D., Sharma A., Sangnim T. (2022). A Comprehensive Review on Nutraceuticals: Therapy Support and Formulation Challenges. Nutrients.

[45] Ciriminna R., Meneguzzo F., Delisi R., Pagliaro M. (2017). Olive Biophenols as New Antioxidant Additives in Food and Beverage. ChemistrySelect.

[46] Gutiérrez-del-Río I., López-Ibáñez S., Magadán-Corpas P., Fernández-Calleja L., Pérez-Valero Á., Tuñón-Granda M., Miguélez E.M., Villar C.J., Lombó F. (2021). Terpenoids and Polyphenols as Natural Antioxidant Agents in Food Preservation. Antioxidants.

[47] Wayan Arnata I., Anggreni A.A.M.D., Arda G., Masruchin N., Sartika D., Fahma F., Firmanda A. (2024). Minimising Food Oxidation Using Aromatic Polymer: From Lignin into Nano-Lignin. Food Res. Int..

[48] Šojić B., Milošević S., Savanović D., Zeković Z., Tomović V., Pavlić B. (2023). Isolation, Bioactive Potential, and Application of Essential Oils and Terpenoid-Rich Extracts as Effective Antioxidant and Antimicrobial Agents in Meat and Meat Products. Molecules.

[49] Al-Refaie D., Mehyar G.F., Shahein M. (2023). Functional Role of Essential Oils as Antimicrobial and Antioxidat Agents in Food Industry: A Review. Jordan J. Agric. Sci..

[50] Hussen N.H.A., Abdulla S.K., Ali N.M., Ahmed V.A., Hasan A.H., Qadir E.E. (2025). Role of Antioxidants in Skin Aging and the Molecular Mechanism of ROS: A Comprehensive Review. Asp. Mol. Med..

[51] Zhang J., Yang Y., Xu H., Li X., Dong F., Chen Q., Han T., Wang J., Wu C. (2024). Effects of Dietary Astaxanthin on Growth Performance, Immunity, and Tissue Composition in Largemouth Bass, Micropterus Salmoides. Front. Mar. Sci..

[52] Li B., Chen C., Zhou X., Liu H., Zhou Z., Wang X., Liang J., Guo Y., Liang S. (2025). Effectiveness of Astaxanthin as a Feed Supplement to Improve Growth Performance and Feed Utilization in Aquaculture Animals: A Meta-Analysis. Antioxidants.

[53] Kumar D., Bhardwaj R., Jassal S., Goyal T., Khullar A., Gupta N. (2021). Application of Enzymes for an Eco-Friendly Approach to Textile Processing. Environ. Sci. Pollut. Res..

[54] Dong J., Chi Z., Lu S., Xie X., Gong P., Li H., Liu W. (2025). Bacterial Exopolysaccharides: Characteristics and Antioxidant Mechanism. Int. J. Biol. Macromol..

[55] Parveen B., Rajinikanth V., Narayanan M. (2025). Natural Plant Antioxidants for Food Preservation and Emerging Trends in Nutraceutical Applications. Discov. Appl. Sci..

[56] Hamidi M., Kozani P.S., Kozani P.S., Pierre G., Michaud P., Delattre C. (2019). Marine Bacteria versus Microalgae: Who Is the Best for Biotechnological Production of Bioactive Compounds with Antioxidant Properties and Other Biological Applications?. Mar. Drugs.

[57] Ma B., Bu Y., Huang J., Liu Y., Guo Z., Yu H., Liang T., Wang D. (2025). Bioactive Compounds from Deep-Sea Extremophiles: Emerging Potential in Cosmeceuticals and Nutraceuticals. FEMS Microbiol. Lett..

[58] Gallo G., Aulitto M. (2024). Advances in Extremophile Research: Biotechnological Applications through Isolation and Identification Techniques. Life.

[59] John G.E., Elijah A.I., Effiong B.N., Okpo E.A., Lennox J.A. (2025). Extremophiles and Their Potentials in the Food Industries: A Review. Asian Food Sci. J..

[60] Marzban G., Tesei D. (2025). The Extremophiles: Adaptation Mechanisms and Biotechnological Applications. Biology.

[61] Rawat M., Chauhan M., Pandey A. (2024). Extremophiles and Their Expanding Biotechnological Applications. Arch. Microbiol..

[62] Uppar A.P., Vanti G.L., Poondla N., Veeresh S., Kaulgud R.S., Kurjogi M.M., Shah M.P., Dey S. (2024). Applications of Extremophiles in Therapeutics. Trends in Biotechnology of Polyextremophiles.

[63] González E., Vera F., Scott F., Guerrero C., Bolívar J.M., Aroca G., Muñoz J.Á., Ladero M., Santos V.E. (2024). Acidophilic Heterotrophs: Basic Aspects and Technological Applications. Front. Microbiol..

[64] Fernández-López M.G., Batista-García R.A., Aréchiga-Carvajal E.T. (2023). Alkaliphilic/Alkali-Tolerant Fungi: Molecular, Biochemical, and Biotechnological Aspects. J. Fungi.

[65] Preiss L., Hicks D.B., Suzuki S., Meier T., Krulwich T.A. (2015). Alkaliphilic Bacteria with Impact on Industrial Applications, Concepts of Early Life Forms, and Bioenergetics of ATP Synthesis. Front. Bioeng. Biotechnol..

[66] Santillan E.-F.U., Shanahan T.M., Omelon C.R., Major J.R., Bennett P.C. (2015). Isolation and Characterization of a CO2-Tolerant Lactobacillus Strain from Crystal Geyser, Utah, U.S.A.. Front. Earth Sci..

[67] Oren A. (2024). Novel Insights into the Diversity of Halophilic Microorganisms and Their Functioning in Hypersaline Ecosystems. Npj Biodivers..

[68] Kanekar P.P., Kanekar S.P. (2022). Metallophilic, Metal-Resistant, and Metal-Tolerant Microorganisms. Diversity and Biotechnology of Extremophilic Microorganisms from India; Microorganisms for Sustainability.

[69] Scoma A. (2021). Functional Groups in Microbial Ecology: Updated Definitions of Piezophiles as Suggested by Hydrostatic Pressure Dependence on Temperature. ISME J..

[70] Sharma D.K., Soni I., Rajpurohit Y.S. (2025). Surviving the Storm: Exploring the Role of Natural Transformation in Nutrition and DNA Repair of Stressed *Deinococcus radiodurans*. Appl. Environ. Microbiol..

[71] Wulandari D., Budiharjo A., Putri A.A.K., Setiarto R.H.B. (2025). Diversity of Thermophilic Bacteria Isolated from Extreme Environments in Indonesia: A Perspective in Biotechnology Applications. J. Trop. Biodivers. Biotechnol..

[72] Pócsi I., Dijksterhuis J., Houbraken J., De Vries R.P. (2024). Biotechnological Potential of Salt Tolerant and Xerophilic Species of Aspergillus. Appl. Microbiol. Biotechnol..

[73] Duran S.K., Kumar P., Sandhu S.S. (2021). A Review on Microalgae Strains, Cultivation, Harvesting, Biodiesel Conversion and Engine Implementation. Biofuels.

[74] Wong J.F., Hong H.J., Foo S.C., Yap M.K.K., Tan J.W. (2022). A Review on Current and Future Advancements for Commercialized Microalgae Species. Food Sci. Hum. Wellness.

[75] Kryvenda A., Tischner R., Steudel B., Griehl C., Armon R., Friedl T. (2023). Testing for Terrestrial and Freshwater Microalgae Productivity under Elevated CO_2_ Conditions and Nutrient Limitation. BMC Plant Biol..

[76] Varshney P., Mikulic P., Vonshak A., Beardall J., Wangikar P.P. (2015). Extremophilic Micro-Algae and Their Potential Contribution in Biotechnology. Bioresour. Technol..

[77] Rinaldi K.L., Senhorinho G.N.A., Laamanen C.A., Scott J.A. (2024). A Review of Extremophilic Microalgae: Impacts of Experimental Cultivation Conditions for the Production of Antimicrobials. Algal Res..

[78] Marchetto F., Conde T., Śliwińska M.A., Rewerski B., Lebiedzińska-Arciszewska M., Szymański J., Więckowski M.R., Matlakowska R., Domingues M.R., Kargul J. (2025). Adaptive Laboratory Evolution of Extremophilic Red Microalga Cyanidioschyzon merolae under High Nickel Stress Enhances Lipid Production and Alleviates Oxidative Damage. Bioresour. Technol..

[79] Miyagishima S.-Y., Tanaka K. (2021). The Unicellular Red Alga *Cyanidioschyzon merolae*—The Simplest Model of a Photosynthetic Eukaryote. Plant Cell Physiol..

[80] Marchetto F., Santaeufemia S., Lebiedzińska-Arciszewska M., Śliwińska M.A., Pich M., Kurek E., Naziębło A., Strawski M., Solymosi D., Szklarczyk M. (2024). Dynamic Adaptation of the Extremophilic Red Microalga Cyanidioschyzon merolae to High Nickel Stress. Plant Physiol. Biochem..

[81] Salbitani G., Perrone A., Rosati L., Laezza C., Carfagna S. (2022). Sulfur Starvation in Extremophilic Microalga Galdieria Sulphuraria: Can Glutathione Contribute to Stress Tolerance?. Plants.

[82] Zheng Y., Xue C., Chen H., He C., Wang Q. (2020). Low-Temperature Adaptation of the Snow Alga Chlamydomonas Nivalis Is Associated with the Photosynthetic System Regulatory Process. Front. Microbiol..

[83] Yao H., Xu Y., Yang H., Guo Y., Jiao P., Xiang D., Xu H., Cao Y. (2025). Glycerol Biosynthesis Pathways from Starch Endow Dunaliella Salina with the Adaptability to Osmotic and Oxidative Effects Caused by Salinity. Int. J. Mol. Sci..

[84] Romero-Cruz M.D.C., Leon-Vaz A., Giráldez I., Vega J.M., Vigara J. (2024). Effect of Heavy Metals on the Antioxidant System of the Acid-Tolerant Microalga Coccomyxa Onubensis. Algal Res..

[85] Hirooka S., Miyagishima S. (2016). Cultivation of Acidophilic Algae Galdieria Sulphuraria and Pseudochlorella Sp. YKT1 in Media Derived from Acidic Hot Springs. Front. Microbiol..

[86] Procházková L., Leya T., Křížková H., Nedbalová L. (2019). Sanguina Nivaloides and Sanguina Aurantia Gen. et Spp. Nov. (Chlorophyta): The Taxonomy, Phylogeny, Biogeography and Ecology of Two Newly Recognised Algae Causing Red and Orange Snow. FEMS Microbiol. Ecol..

[87] Chekanov K. (2023). Diversity and Distribution of Carotenogenic Algae in Europe: A Review. Mar. Drugs.

[88] Pasqualetti M., Tempesta S., Malavasi V., Barghini P., Fenise M. (2015). Lutein Production by Coccomyxa Sp. SCCA048 Isolated from a Heavy Metal-Polluted River in Sardinia (Italy). J. Environ. Prot. Ecol..

[89] Cuaresma M., Casal C., Forján E., Vílchez C. (2011). Productivity and Selective Accumulation of Carotenoids of the Novel Extremophile Microalga Chlamydomonas Acidophila Grown with Different Carbon Sources in Batch Systems. J. Ind. Microbiol. Biotechnol..

[90] Bermejo E., Ruiz-Domínguez M.C., Cuaresma M., Vaquero I., Ramos-Merchante A., Vega J.M., Vílchez C., Garbayo I. (2018). Production of Lutein, and Polyunsaturated Fatty Acids by the Acidophilic Eukaryotic Microalga Coccomyxa Onubensis under Abiotic Stress by Salt or Ultraviolet Light. J. Biosci. Bioeng..

[91] Fuentes J.-L., Montero Z., Cuaresma M., Ruiz-Domínguez M.-C., Mogedas B., Nores I.G., González Del Valle M., Vílchez C. (2020). Outdoor Large-Scale Cultivation of the Acidophilic Microalga Coccomyxa Onubensis in a Vertical Close Photobioreactor for Lutein Production. Processes.

[92] Chu X., Liu J., Gu W., Tian L., Tang S., Zhang Z., Jiang L., Xu X. (2022). Study of the Properties of Carotenoids and Key Carotenoid Biosynthesis Genes from *Deinococcus xibeiensis* R13. Biotechnol. Appl. Biochem..

[93] Wan M., Zhao H., Guo J., Yan L., Zhang D., Bai W., Li Y. (2021). Comparison of C-Phycocyanin from Extremophilic Galdieria Sulphuraria and Spirulina Platensis on Stability and Antioxidant Capacity. Algal Res..

[94] Gammoudi S., Dahmen-Ben Moussa I., Annabi-Trabelsi N., Ayadi H., Guermazi W., Waisundara V. (2021). Antioxidant Properties of Metabolites from New Extremophiles Microalgal Strain (Southern, Tunisia). Antioxidants—Benefits, Sources, Mechanisms of Action.

[95] León-Vaz A., León R., Vigara J., Funk C. (2023). Exploring Nordic Microalgae as a Potential Novel Source of Antioxidant and Bioactive Compounds. New Biotechnol..

[96] Espina G., Atalah J., Blamey J.M. (2021). Extremophilic Oxidoreductases for the Industry: Five Successful Examples With Promising Projections. Front. Bioeng. Biotechnol..

[97] Liu J., Yin M., Zhu H., Lu J., Cui Z. (2011). Purification and Characterization of a Hyperthermostable Mn-Superoxide Dismutase from Thermus Thermophilus HB27. Extremophiles.

[98] Wang Q., Nie P., Hou Y., Wang Y. (2020). Purification, Biochemical Characterization and DNA Protection against Oxidative Damage of a Novel Recombinant Superoxide Dismutase from Psychrophilic Bacterium Halomonas Sp. ANT108. Protein Expr. Purif..

[99] Sartorio M.G., Cortez N., González J.M. (2021). Structure and Functional Properties of the Cold-Adapted Catalase from *Acinetobacter* Sp. Ver3 Native to the Atacama Plateau in Northern Argentina. Acta Crystallogr. Sect. Struct. Biol..

[100] Monsalves M.T., Ollivet-Besson G.P., Amenabar M.J., Blamey J.M. (2020). Isolation of a Psychrotolerant and UV-C-Resistant Bacterium from Elephant Island, Antarctica with a Highly Thermoactive and Thermostable Catalase. Microorganisms.

[101] Egorenko M.Y., Baiguzhin G.F., Kiseleva M.A., Gorokhovskaya I.N., Shmelin P.S. (2022). Cultivation of the Halobacterium Salinarum Biomass with High Antioxidant Activity for Agricultural and Food Industry. IOP Conf. Ser. Earth Environ. Sci..

[102] Squillaci G., Parrella R., Carbone V., Minasi P., La Cara F., Morana A. (2017). Carotenoids from the Extreme Halophilic Archaeon Haloterrigena Turkmenica: Identification and Antioxidant Activity. Extremophiles.

[103] Tapia C., López B., Astuya A., Becerra J., Gugliandolo C., Parra B., Martínez M. (2021). Antiproliferative Activity of Carotenoid Pigments Produced by Extremophile Bacteria. Nat. Prod. Res..

[104] Sadowska-Bartosz I., Bartosz G. (2023). Antioxidant Defense of *Deinococcus radiodurans*: How Does It Contribute to Extreme Radiation Resistance?. Int. J. Radiat. Biol..

[105] Lim S., Song H.-Y., Park H.R., Ahn K.B. (2024). A Novel Deinococcus Antioxidant Peptide Mitigates Oxidative Stress in Irradiated CHO-K1 Cells. Microorganisms.

[106] Furukawa Y., Megata M., Shintani A., Sue K., Morohoshi T., Akutsu M., Muraki N. (2025). Cu/Zn-Superoxide Dismutase Naturally Fused with a β-Propeller Lactonase in Deinococcus radiodurans. J. Biol. Chem..

[107] Pathak J., Ahmed H., Rajneesh, Singh S.P., Häder D.-P., Sinha R.P. (2019). Genetic Regulation of Scytonemin and Mycosporine-like Amino Acids (MAAs) Biosynthesis in Cyanobacteria. Plant Gene.

[108] Ručová D., Vilková M., Sovová S., Vargová Z., Kostecká Z., Frenák R., Routray D., Bačkor M. (2023). Photoprotective and Antioxidant Properties of Scytonemin Isolated from Antarctic Cyanobacterium Nostoc Commune Vaucher Ex Bornet & Flahault and Its Potential as Sunscreen Ingredient. J. Appl. Phycol..

[109] Kokabi M., Yousefzadi M., Soltani M., Arman M. (2019). Effects of Different UV Radiation on Photoprotective Pigments and Antioxidant Activity of the Hot-spring Cyanobacterium *Leptolyngbya* Cf. *Fragilis*. Phycol. Res..

[110] Orellana G., Gómez-Silva B., Urrutia M., Galetović A. (2020). UV-A Irradiation Increases Scytonemin Biosynthesis in Cyanobacteria Inhabiting Halites at Salar Grande, Atacama Desert. Microorganisms.

[111] Casero M.C., Herrero M.Á., De La Roche J.P., Quesada A., Velázquez D., Cirés S. (2024). Effect of Salinity on Scytonemin Yield in Endolithic Cyanobacteria from the Atacama Desert. Sci. Rep..

[112] George A.L., Murray A.W., Montiel P.O. (2001). Tolerance of Antarctic Cyanobacterial Mats to Enhanced UV Radiation. FEMS Microbiol. Ecol..

[113] Dishliyska V., Stoyancheva G., Abrashev R., Miteva-Staleva J., Spasova B., Angelova M., Krumova E. (2023). Catalase from the Antarctic Fungus Aspergillus Fumigatus I-9–Biosynthesis and Gene Characterization. Indian J. Microbiol..

[114] Koleva Z., Abrashev R., Angelova M., Stoyancheva G., Spassova B., Yovchevska L., Dishliyska V., Miteva-Staleva J., Krumova E. (2024). A Novel Extracellular Catalase Produced by the Antarctic Filamentous Fungus Penicillium Rubens III11-2. Fermentation.

[115] Abrashev R., Feller G., Kostadinova N., Krumova E., Alexieva Z., Gerginova M., Spasova B., Miteva-Staleva J., Vassilev S., Angelova M. (2016). Production, Purification, and Characterization of a Novel Cold-Active Superoxide Dismutase from the Antarctic Strain Aspergillus Glaucus 363. Fungal Biol..

[116] Pacelli C., Cassaro A., Maturilli A., Timperio A.M., Gevi F., Cavalazzi B., Stefan M., Ghica D., Onofri S. (2020). Multidisciplinary Characterization of Melanin Pigments from the Black Fungus Cryomyces Antarcticus. Appl. Microbiol. Biotechnol..

[117] Catanzaro I., Gorbushina A.A., Onofri S., Schumacher J. (2024). 1,8-Dihydroxynaphthalene (DHN) Melanin Provides Unequal Protection to Black Fungi *Knufia petricola* and *Cryomyces antarcticus* from UV—B Radiation. Environ. Microbiol. Rep..

[118] De León L.R., Moreno-Perlín T., Castillo-Marenco T., Del Rayo Sánchez-Carbente M., Gostinčar C., Ramírez-Durán N., Ocaña A.M.F., Sánchez N.C., Dávila-Ramos S., Gunde-Cimerman N. (2025). Polyextremotolerant, Opportunistic, and Melanin-Driven Resilient Black Yeast Exophiala Dermatitidis in Environmental and Clinical Contexts. Sci. Rep..

[119] Flieger K., Knabe N., Toepel J. (2018). Development of an Improved Carotenoid Extraction Method to Characterize the Carotenoid Composition under Oxidative Stress and Cold Temperature in the Rock Inhabiting Fungus Knufia Petricola A95. J. Fungi.

[120] De Menezes G.C.A., De Medeiros T.D.M., De Oliveira Lima I.G., Da Silva M.B., De Queiroz A.C., Duarte A.W.F., De Oliveira V.M., Rosa L.H., Bicas J.L., Chhikara N., Panghal A., Chaudhary G. (2023). Pigments Produced by Fungi and Bacteria from Extreme Environments. Microbes in the Food Industry.

[121] Śliżewska W., Struszczyk-Świta K., Marchut-Mikołajczyk O. (2022). Metabolic Potential of Halophilic Filamentous Fungi—Current Perspective. Int. J. Mol. Sci..

[122] Trochine A., Turchetti B., Vaz A.B.M., Brandao L., Rosa L.H., Buzzini P., Rosa C., Libkind D. (2017). Description of Dioszegia Patagonica Sp. Nov., a Novel Carotenogenic Yeast Isolated from Cold Environments. Int. J. Syst. Evol. Microbiol..

[123] Cavalcante S.B., Dos Santos Biscaino C., Kreusch M.G., Da Silva A.F., Duarte R.T.D., Robl D. (2023). The Hidden Rainbow: The Extensive Biotechnological Potential of Antarctic Fungi Pigments. Braz. J. Microbiol..

[124] Sakaki H., Kaneno H., Sumiya Y., Tsushima M., Miki W., Kishimoto N., Fujita T., Matsumoto S., Komemushi S., Sawabe A. (2002). A New Carotenoid Glycosyl Ester Isolated from a Marine Microorganism, *Fusarium* Strain T-1. J. Nat. Prod..

[125] Bannister J.V., Bannister W.H., Rotilio G. (1987). Aspects of the Structure, Function, and Applications of Superoxide Dismutas. Crit. Rev. Biochem..

[126] Steimbrüch B.A., Sartorio M.G., Cortez N., Albanesi D., Lisa M.-N., Repizo G.D. (2022). The Distinctive Roles Played by the Superoxide Dismutases of the Extremophile Acinetobacter Sp. Ver3. Sci. Rep..

[127] Dong X., Wang W., Li S., Han H., Lv P., Yang C. (2021). Thermoacidophilic Alicyclobacillus Superoxide Dismutase: Good Candidate as Additives in Food and Medicine. Front. Microbiol..

[128] Thakur A., Kumar P., Kumari P., Bhatia K., Chand D. (2020). Statistical Augmentation of Thermostable Superoxide Dismutase (SOD) Production from Bacillus Licheniformis SPB-13 of Himalayan Ranges. Afr. J. Biol. Sci..

[129] Hamre A.G., Al-Sadawi R., Johannesen K.M., Bisarro B., Kjendseth Å.R., Leiros H.-K.S., Sørlie M. (2023). Initial Characterization of an Iron Superoxide Dismutase from Thermobifida Fusca. JBIC J. Biol. Inorg. Chem..

[130] Mandelli F., Miranda V.S., Rodrigues E., Mercadante A.Z. (2012). Identification of Carotenoids with High Antioxidant Capacity Produced by Extremophile Microorganisms. World J. Microbiol. Biotechnol..

[131] Waditee-Sirisattha R., Kageyama H. (2022). Extremophilic Cyanobacteria. Cyanobacterial Physiology.

[132] Gostinčar C., Stajich J.E., Gunde-Cimerman N. (2023). Extremophilic and Extremotolerant Fungi. Curr. Biol..

[133] Lyall R., Nikoloski Z., Gechev T. (2020). Comparative Analysis of ROS Network Genes in Extremophile Eukaryotes. Int. J. Mol. Sci..

[134] Toledo A.V., Franco M.E.E., Yanil Lopez S.M., Troncozo M.I., Saparrat M.C.N., Balatti P.A. (2017). Melanins in Fungi: Types, Localization and Putative Biological Roles. Physiol. Mol. Plant Pathol..

[135] Bartolewska M., Kosik-Kozioł A., Arif A., Nakielski P., Pierini F. (2026). Natural Melanin: A Multifunctional Biopigment for Advanced Biomedical Applications. Prog. Biomed. Eng..

[136] Robinson C.H. (2001). Cold Adaptation in Arctic and Antarctic Fungi. New Phytol..

[137] Cecchi T., Pezzella A., Di Mauro E., Cestola S., Ginsburg D., Luzi M., Rigucci A., Santato C. (2020). On the Antioxidant Activity of Eumelanin Biopigments: A Quantitative Comparison between Free Radical Scavenging and Redox Properties. Nat. Prod. Res..

[138] Mavridi-Printezi A., Menichetti A., Mordini D., Amorati R., Montalti M. (2023). Recent Applications of Melanin-like Nanoparticles as Antioxidant Agents. Antioxidants.

[139] Suthar M., Dufossé L., Singh S.K. (2023). The Enigmatic World of Fungal Melanin: A Comprehensive Review. J. Fungi.

[140] Mansour R.I., Elshoubaky G.A., Attia E.A. (2025). Exploring Antioxidant and Cytotoxicity Prospective of Melanin Pigment Produced by Fungi Associated with Marine Macroalgae. Microb. Biosyst..

[141] Pal A.K., Gajjar D.U., Vasavada A.R. (2013). DOPA and DHN Pathway Orchestrate Melanin Synthesis in *Aspergillus* Species. Med. Mycol..

[142] Medina-Armijo C., Yousef I., Berná A., Puerta A., Esteve-Núñez A., Viñas M., Prenafeta-Boldú F.X. (2024). Characterization of Melanin from Exophiala Mesophila with the Prospect of Potential Biotechnological Applications. Front. Fungal Biol..

[143] Gunde-Cimerman N., Plemenitaš A., Oren A. (2018). Strategies of Adaptation of Microorganisms of the Three Domains of Life to High Salt Concentrations. FEMS Microbiol. Rev..

[144] Miller A.-F. (2012). Superoxide Dismutases: Ancient Enzymes and New Insights. FEBS Lett..

[145] Gogliettino M., Arciello S., Cillo F., Carluccio A.V., Palmieri G., Apone F., Ambrosio R.L., Anastasio A., Gratino L., Carola A. (2022). Recombinant Expression of Archaeal Superoxide Dismutases in Plant Cell Cultures: A Sustainable Solution with Potential Application in the Food Industry. Antioxidants.

[146] Ibrahim N.E., Ma K. (2017). Industrial Applications of Thermostable Enzymes from Extremophilic Microorganisms. Curr. Biochem. Eng..

[147] Takio N., Yadav M., Yadav H.S. (2021). Catalase-Mediated Remediation of Environmental Pollutants and Potential Application—A Review. Biocatal. Biotransformation.

[148] Espina G., Muñoz-Ibacache S.A., Cáceres-Moreno P., Amenabar M.J., Blamey J.M. (2022). From the Discovery of Extremozymes to an Enzymatic Product: Roadmap Based on Their Applications. Front. Bioeng. Biotechnol..

[149] Lugani Y., Vemuluri V.R. (2022). Extremophiles Diversity, Biotechnological Applications and Current Trends. Extremophiles.

[150] Ashaolu T.J., Malik T., Soni R., Prieto M.A., Jafari S.M. (2025). Extremophilic Microorganisms as a Source of Emerging Enzymes for the Food Industry: A Review. Food Sci. Nutr..

[151] Yan X., Xu Y., Wei T., Chai Y., Li Y., Wang C., Li M., Zhang S., Zhu W., Liu Z. (2024). Modified Chitosan for Highly Efficient Non-Invasive Transdermal Delivery of Catalase to Repair and Prevent Skin Photodamages. Adv. Funct. Mater..

[152] Gong M., Bassi A. (2016). Carotenoids from Microalgae: A Review of Recent Developments. Biotechnol. Adv..

[153] Öztürk S., Satpati G.G., Shah M.P., Dey S. (2024). Extremophilic Algae-Based Wastewater Treatment, Nutrient Recovery, and Animal Feed Production. Trends in Biotechnology of Polyextremophiles.

[154] Ambati R., Phang S.-M., Ravi S., Aswathanarayana R. (2014). Astaxanthin: Sources, Extraction, Stability, Biological Activities and Its Commercial Applications—A Review. Mar. Drugs.

[155] Li J., Zhu D., Niu J., Shen S., Wang G. (2011). An Economic Assessment of Astaxanthin Production by Large Scale Cultivation of Haematococcus Pluvialis. Biotechnol. Adv..

[156] Saikia D.K., Ahmed R., Chikkaputtaiah C., Velmurugan N., Raja R., Hemaiswarya S., Narayanan M., Kandasamy S., Jayappriyan K.R. (2023). Commercialization of Haematococcus-Based Products: Current Status and Future Forecast. Haematococcus.

[157] Grewe C.B., Griehl C. (2012). 8 The carotenoid astaxanthin from Haematococcus pluvialis. Microalgal Biotechnol. Integr. Econ..

[158] Shah M.M.R., Liang Y., Cheng J.J., Daroch M. (2016). Astaxanthin-Producing Green Microalga Haematococcus Pluvialis: From Single Cell to High Value Commercial Products. Front. Plant Sci..

[159] Spolaore P., Joannis-Cassan C., Duran E., Isambert A. (2006). Commercial Applications of Microalgae. J. Biosci. Bioeng..

[160] Askre D., Ohta Y. (2002). Production of Canthaxanthin by Haloferax Alexandrinus under Non-Aseptic Conditions and a Simple, Rapid Method for Its Extraction. Appl. Microbiol. Biotechnol..

[161] Coker J.A. (2016). Extremophiles and Biotechnology: Current Uses and Prospects. F1000Research.

